# The Quest for an Alzheimer Therapy

**DOI:** 10.3389/fneur.2018.00108

**Published:** 2018-03-01

**Authors:** Stefano F. Cappa

**Affiliations:** ^1^Institute for Advanced Studies (IUSS), Pavia, Italy; ^2^IRCCS S. Giovanni di Dio Fatebenefratelli, Brescia, Italy

**Keywords:** Alzheimer disease, dementia prevention, amyloid, therapy, diagnostic criteria

## Abstract

This mini-review considers three different approaches to the therapy and prevention of Alzheimer’s disease (AD): replacement therapy, disease modification, and multi-level interventions. Each of these research frameworks has direct implications at the clinical level, leading to an emphasis on different time points of the AD continuum. While all perspectives continue to play an important role in current efforts to reach the ambitious target of an effective therapy or prevention of AD by 2025, it is clear that novel paradigms are needed, including new models of clinical trial design. This goal can only be accomplished by a concerted effort of academia, governmental agencies, and industry.

## Introduction

The increasing awareness that dementia, and in particular Alzheimer’s disease (AD), represents one of the major challenges to health systems in coming years has led to an unprecedented emphasis on the need for an effective therapy, now considered as a priority for science and society (1). The year 2025 has been set by world leaders as the target for the availability of an effective therapy or prevention of AD (2).

The modern history of research for an AD therapy can be conceptualized in three different approaches, which are briefly discussed in this review: replacement therapy, disease modification, and multi-level intervention. These approaches cannot be conceived as stages in an evolutionary process, since all of them are still playing a central role in current research. It must be, however, underlined that they have different implications for the process of diagnosing AD, as manifested by the progressive changes in diagnostic criteria, and guidelines that have taken place in the past decades, from the NINCDS-ADRDA Work Group (3), to the current research framework promoted by National Institute on Aging and Alzheimer’s Association (NIA-AA) with the aim of updating and unifying the most recent published set of diagnostic criteria (4, 5).

## Replacement Therapy for Cognitive Dysfunction

The successful introduction of l-DOPA for Parkinson’s disease had a clear impact on the AD therapy research field. The concept that disease manifestations could be defined in terms of neurotransmitter loss was a central component of the cholinergic hypothesis, and led to the development of the leading class of drugs currently approved for AD therapy, i.e., acetylcholinesterase (AChe) inhibitor drugs. The systematic search for a biochemical fingerprint for AD began in the 1960s of past century, and was crowned by success with the discovery of reduced level of choline acetyltransferase, the enzyme responsible for ACh synthesis, in the cortex of AD patients (6), soon linked to neural loss in the nucleus basalis of Meynert (7). These findings opened the way to the search of possible “replacement” therapies, clearly modeled on the PD approach (8), and based on the prediction that drugs increasing cholinergic neurotransmission in the AD brain could be expected to result in a symptomatic treatment of the cognitive hallmark of AD, i.e., memory dysfunction. This concept was additionally supported by the evidence of a dysmnestic effect of anticholinergic drugs, such as scopolamine, in experimental and human studies (9). After some disappointments due to the failure of increase central ACh by direct administration of ACh precursors, the era of AChEI was opened by the tacrine trials. An excellent review of the state-of-the-art at the end of the past century can be found in Ref. (10), where the limits of the “replacement” approach are also acknowledged. Attempts to extend the neurotransmitter replacement idea to other molecules potentially associated with specific aspects of cognitive dysfunction (e.g., noradrenaline and attention) also met with limited success (11).

At the diagnostic level, this approach is necessarily linked to the identification of the presence of cognitive deficits, possibly reflecting specific neurotransmitter dysfunctions, which can become the target of a specific replacement therapy. The NINCDS-ADRDA criteria (3), which remained the standard diagnostic reference in the field of AD for more than two decades, require the presence of dementia for the definition of probable AD. The diagnostic process delineated in these criteria is purely clinical/neuropsychological, and the “ancillary examinations” are used to provide exclusionary evidence for other possible causes of the dementia syndrome.

The need for symptomatic treatments based on neurotransmitter mechanisms is not limited to cognitive enhancement, but includes the clinically crucial aspect of control of neuropsychiatric symptoms, the main determinant of burden of care in AD patients (12). In a recent review of current clinical trials in AD (13), 14% of the drugs under trial are symptomatic cognitive enhancers, and 13% are symptomatic agents addressing neuropsychiatric and behavioral changes. Most of these drugs have mechanisms of action involving different neurotransmitter systems, such as 5-HT or cannabinoids, indicating the continuing role of this approach, in particular at the level of dementia care (14).

## The Holy Grail of Disease Modification

**Figure 1:**
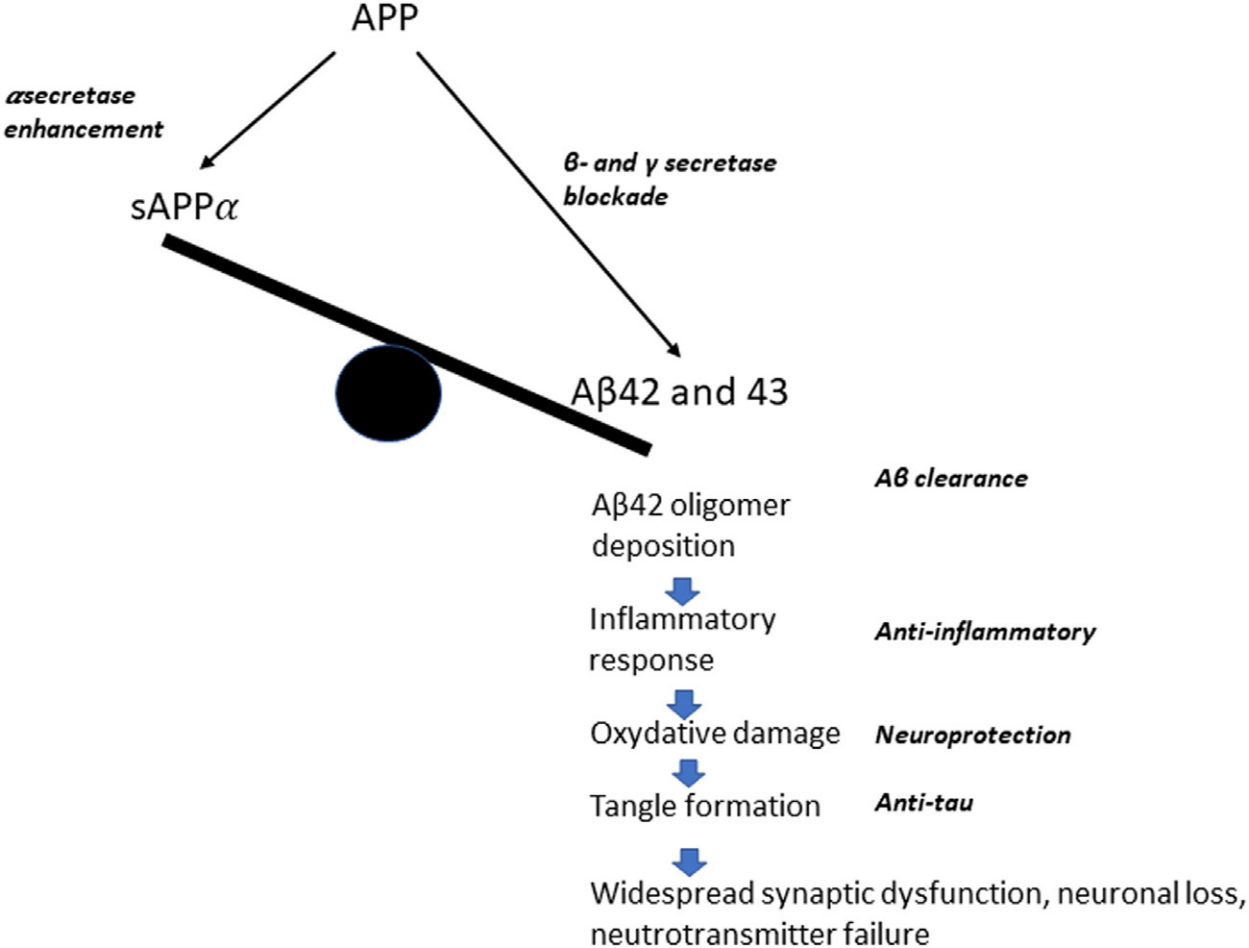
A simplified diagram of the possible therapeutic approaches, according to the amyloid cascade hypothesis.

The “modern era” of AD research is closely linked to the birth of the amyloid hypothesis of the pathogenesis of disease in the early 1990s. The foundations of this hypothesis were provided by the discovery of amyloid as the main component of the senile plaques and of the pathogenetic role of amyloid precursor protein (APP) mutations [see in Ref. (15) for an early review]. These findings promoted a change of focus from the condition of established dementia to the early stages of disease, which could represent a target for interventions whose ambition was the modification of the disease process, rather than a replacement of the consequences of brain damage. The search for a disease-modifying treatment (DMT) for Alzheimer’s disease (AD) was motivated by “advances in the understanding of neurodegenerative mechanisms in Alzheimer’s disease” in areas, such as neurotrophic factors, protein processing, oxidative stress, and inflammatory processes (16). The main focus of DMT approaches has centered on the amyloid cascade hypothesis [ACH—for an updated review, see in Ref. (17)]. Several different therapeutic approaches have been developed within this conceptual framework (Figure 1), which attributes a central role in neuronal degeneration and cognitive decline to the pathological aggregation of amyloid β (Aβ) peptides, predominantly Aβ_40_ and Aβ_42_. Aβ is derived from APP *via* proteolysis by two enzyme complexes, β-secretase and γ-secretase. While the alternative pathway, mediated by α-secretase, results in the production of a soluble sAPPα fragment, under pathological conditions the soluble oligomers of Aβ_42_ result in synaptic dysfunction (decrease in synapse number, inhibition of long-term potentiation, and enhancement of long-term synaptic depression), starting a complex chain of molecular events and cellular reactions finally leading to plaque formation and neuronal death [for an updated review, see in Ref. (18)]. A model of these pathological conditions was provided by the rare dominantly inherited forms of AD, where missense mutations in the APP or presenilin 1 or 2 genes lead to a lifelong increase of Aβ_42_ and Aβ_43_ production, i.e., longer, more hydrophobic forms than Aβ_40_, which, even in low amounts, may have higher neurotoxic potential (17). The original formulation of the amyloid cascade hypothesis proposed that “accumulation of Aβ in the brain is the primary influence driving AD pathogenesis. The rest of the disease process, including formation of neurofibrillary tangles containing tau protein, is proposed to result from an imbalance between Aβ production and Aβ clearance” (15). The possible therapeutic approaches could then be targeted toward increasing Aβ clearance or modulating its production. The first application of the hypothesis aimed at Aβ clearance, *via* an active immunization approach. The clinical trial with the AN 1792 vaccine was successful in enhancing the production of Aβ antibodies, but failed to show any clinical effect and had to be stopped because of the occurrence of serious cases of meningoencephalitis in treated patients (19). The Aβ clearance approach has remained dominant, and includes both active (vaccine) and passive (monoclonal antibodies) immunization. The latter approach has been extensively pursued in the past decade, with systemic infusion of monoclonal Abs (mAbs) aiming at preventing oligomerization and fibril formation and dissolving Aβ aggregates [for a review, see in Ref. (20)]. The failure of several large-scale trials has generated some legitimate disappointment, but has also provided important lessons about the need to carefully consider the mechanisms of action of different mAbs and the target population (see below). Several trials with newer agents, such as the fully human IgG1 mAb aducanumab (Biogen, Inc.), are ongoing and are supported by positive preliminary evidence of biological efficacy (21). An alternative approach within the same conceptual framework is based on drugs aiming at the modulation of beta amyloid production, with drugs targeting the gamma secretase complex to decrease Aβ production. The relevance of side effects has resulted in many failures to complete the studies, and has led to an increasing interest in the inhibition or modulation of beta secretase, with β-site APP cleaving enzyme 1 (BACE1) as a favorite target (20). The ACH-based therapeutic framework is completed by drugs promoting Aβ degradation (Aβ-degrading proteases) and inhibiting amyloid aggregation (chaperones) (20). A less explored approach is the activation of alpha secretase, with the rationale to stimulate the production of soluble (“good”) APP, which has been attempted with a synthetic retinoid, acitretin (22).

Overall, the appraisal of the results of trials based on the ACH is at the moment negative. The so-called “tau hypothesis” is often considered as a competing approach, but it may be remarked that it shares most of the conceptual assumptions of the amyloid approach, i.e., the idea that the development of AD could be stopped or delayed by interfering with a primary pathological event, i.e., in this case the formation of neurofibrillary tangles. The development of drugs targeting tau aggregation has proven more challenging than in the case of β amyloid, and only recently drugs targeting tau aggregation and of active and passive immunization approaches have entered the clinical trial arena. Among the current clinical trials of DMT, 14 address amyloid targets and only 4 involve tau-related targets (13).

The search for a “magic bullet,” which could halt or slow down the pathological process whose result is the clinical picture of dementia, has had a fundamental impact at the clinical level. Considering dementia as the irreversible outcome of a linear sequence of events unleashed by pathological protein aggregation inevitably leads to the consideration that pathophysiological process leading to dementia begins many years prior to overt clinical manifestations of disease. An early step in this direction was the definition of a “pre-dementia” stage of AD, mild cognitive impairment (23). The following steps were the quest for biomarkers, independent from the presence of clinical symptoms, sufficient to detect the presence of brain pathology. This was an important shift of focus for the diagnostic process. Since, cognitive and functional impairment could not be considered as an effective clinical endpoint for therapeutic trial, the impact of new drugs on AD pathology, the need for human disease biomarkers, derived from structural, functional, and molecular neuroimaging, as well as from neurochemical and genetic studies of AD was widely acknowledged (24). This change of perspective was instrumental in the development of new diagnostic criteria: the International Working Group Research Criteria (25–27), and the National Institute on Aging—Alzheimer’s Association workgroups on diagnostic guidelines (5). The shared concept by these guidelines, i.e., the AD continuum, emphasizes the role of biomarkers in supporting the diagnosis of AD at the very early clinical stages, i.e., when the patient is symptomatic, but does not fulfill the criteria for dementia (prodromal AD, or MCI due to AD). The first step of the diagnostic process, a true gateway to the application of biomarkers, is the presence of memory impairment, defined on the basis of specific tests assessing delayed recall impairment due to medial temporal lobe dysfunction (28). The biomarkers in use aim at the *in vivo* assessment of markers of pathophysiology [amyloid positron emission tomography (PET), low cerebrospinal fluid (CSF) Aβ_42_, and elevated phosphorylated tau (P-tau) and neurodegeneration (AD pattern of FDG PET hypometabolism and hippocampal atrophy on MRI)]. The series of substantial failures of drugs acting on the ACH has been largely attributed to the failure to address the disease process at the earliest possible stages, i.e., before neurodegeneration has taken place. The potential of this approach to early disease stages is now actively investigated in several clinical trials and the results will become available in the next few years.

## Multi-Level Interventions

The molecular and mechanistic views described in the previous section maintain their crucial role in the quest for a therapy of AD, and more encouraging results of the anti-amyloid (and anti-tau) approaches may be forthcoming. It is, however, now clearly acknowledged that the “neuron-centric, linear cascade initiated by Ab and leading to dementia” (18), implying direct causation, needs a revision. AD is a brain disorder, which needs to be investigated at all the multiple levels (cells, networks, computations) intervening between genes and molecules and the clinical phenotype (29). At the molecular and cellular level, this is now acknowledged by approaches considering the complex interplay of neuronal changes with responses of astrocytes, microglia, and of the vascular compartment (18). An increased consideration of the contributing role of neuroinflammation, metabolic modifications, including stress reaction and of neuroprotective agents is testified by the presence of 25% of drugs involving these mechanisms of action under investigation in current clinical trials (2). The emphasis on a long-term chain of feedforward and feedback cellular reactions taking place during the years corresponding to the prodromal phase of AD is in full agreement with the concept of neurodegenerative disorders as progressive dysfunctions of brain-system-specific connectivity (30). While advances in neuroimaging techniques have been a major impulse toward the development of this concept (31), important complementary approaches are emerging from the field of neurophysiology, including the investigation of network-level neural activity changes, such as hypersynchrony and altered rhythmic oscillatory activity, possibly related to interneuronal dysfunction (32), in AD brain. The therapeutic implications of this approach are now starting to be considered (33). The quest for effective drugs targeting protein accumulation can be integrated in a multimodal perspective, considering different levels of intervention at the same time, including treating inflammatory reactions, modulating network dysfunction with invasive and non-invasive neurostimulation, and acting on cognitive dysfunction with lifestyle interventions and cognitive training. The latter aspect has been extensively investigated in the past decades. A recent, outstanding review by Livingston et al. (14) concluded that about 35% of dementia is attributable to a combination of nine risk factors: low educational level, midlife hypertension and obesity, hearing loss, late-life depression, diabetes, physical inactivity, smoking, and social isolation. The concept of cognitive/brain reserve is based on solid experimental evidence of protective effects on cognitive decline and dementia for physical activity, Mediterranean diet, cognitive training, and social engagement (34). The possibility to act positively on the reserve by means of active intervention is suggested by cognitive training studies in healthy elderly subjects, indicating a significant reduction of physiological cognitive decline with working memory training (35). The positive effect of training was not limited to test performance, but extended to functional activities of daily living (36). The FINGER study in at-risk subjects, based on a comprehensive intervention on risk factors combined with cognitive training, reported significant effects on several cognitive variables (37). The multi-dimensional perspective, with its focus of modification of factors increasing and decreasing the risk of cognitive decline, has an inevitable impact on the target population for intervention studies. The focus is moving from the very early clinical stages, characterized by mild symptoms, to asymptomatic at-risk subjects. Preclinical AD trials aim at finding treatments that can postpone, reduce the risk of, or completely prevent the clinical onset of AD (38, 39). The role of biomarkers at this point becomes central, as clinical variables are considered as late markers of disease progression.

## Conclusion

Much has changed in the two decades in the quest for an effective therapy and prevention of AD. The success rate of drug development for AD has been poor and novel paradigms are needed, including new models of clinical trial design taking into account the advances in early diagnosis, the role of genetic factors, and new epidemiological evidence. This can only be accomplished by a concerted effort, including governmental agencies, academic researchers, and industry (1).

## Author Contributions

The author confirms being the sole contributor of this work and approved it for publication.

## Conflict of Interest Statement

The author declares that the research was conducted in the absence of any commercial or financial relationships that could be construed as a potential conflict of interest.
